# Supplementary material for: Novel Long Noncoding RNA miR205HG Functions as an Esophageal Tumor-Suppressive Hedgehog Inhibitor
Source: Cancers (Basel). 2021 Apr 3;13(7):1707. doi: 10.3390/cancers13071707 (PMC8038513; doi:10.3390/cancers13071707)
Supplement: Supplementary file 1 [file cancers-13-01707-s001.pdf]

SUPPLEMENTARY INFORMATION

Novel Long Non-Coding RNA *miR205HG* Functions as An Esophageal Tumor-Suppressive Hedgehog Inhibitor

Jee Hoon Song[1,2*], Alan H. Tieu[1,3*],Yulan Cheng[1*], Ke Ma[1], Venkata S. Akshintala[1], Cem Simsek[1], Vishnu Prasath[1], Eun Ji Shin[1], Saowanee Ngamruengphong[1], Mouen A. Khashab[1], John M. Abraham[1], Stephen J. Meltzer[1]

Table of contents

Supplementary Materials and Methods	Page 2-14
Supplementary Source Supplementary	Page 15-16
Figures	Page 17-19

Supplementary Materials and Methods

OE_33 Cell Line

SKGT4 Cell Line

JHU-Eso-Ad1 Cell Line

Flo-1 Cell Line

QHTRT Cell Line

GiHTRT Cell Line

grcf at Johns Hopkins DNA Services/FAF
Laura Kasch
Johns Hopkins University
2760 Light House Point East, Suite 201
Baltimore, MD 21224
Email: lkasch@jhmi.edu
https://grcf.jhmi.edu/

JOHNS HOPKINS MEDICINE

Cell Line Short Tandem Repeat (STR) Profile Report

Principal Investigator: Yulan Cheng
Lab Group: Meltzer
Institution: Johns Hopkins University, GI
Cell Line ID: oe33
Report Date: November 5, 2018
Submitting Researcher: Yulan Cheng
Sample Type Submitted: cultured cells
GeneSifter Tracking ID: 150777
Profile Kit Requested: GenePrint 10 (Promega)

STR Profile:

Loci	oe33
AMEL	X
CSF1PO	10,11
D13S317	14
D16S539	12
D21S11	29,31.2
D5S818	11
D7S820	9,10
TH01	7,8
TPOX	8,11
vWA	14,17

» Loci with one allele listed are homozygous for that STR marker.

e-Signature Director of Laboratory:

STR Graphic Profile:

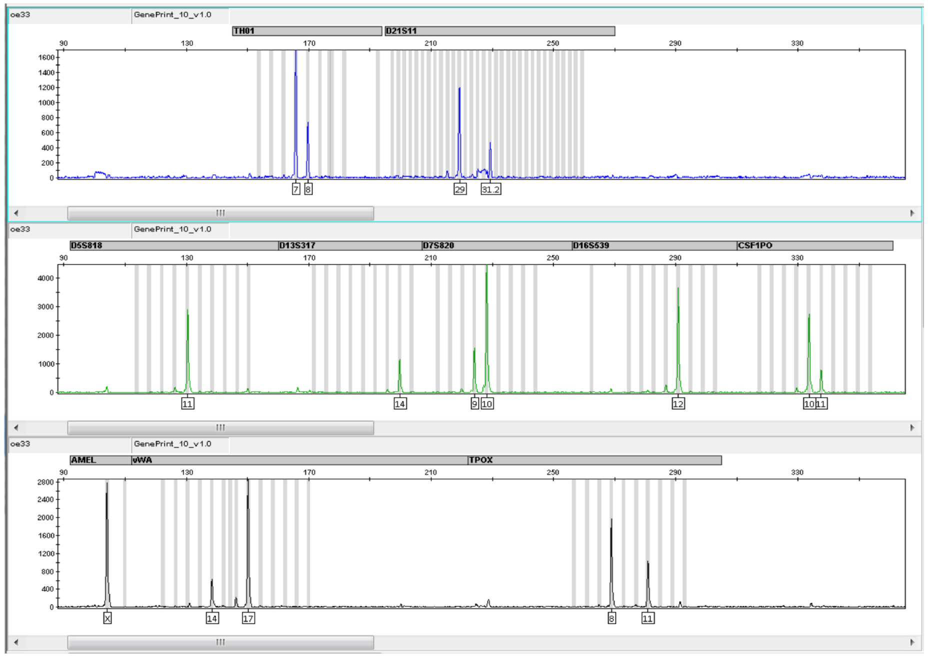

Peaks that appear to be legitimate stutter or are spectral bleed-through artifacts are not labeled.

- This report provides a STR profile for the cell line submitted, it does not automatically authenticate the cell line. Authentication is determined by comparison to the STR profile of the original donor tissue or repository cell stock. This STR profile can be used to verify the cell line is human, evaluate profile consistency between provisionally related cell isolates, detect cross-contamination with another human cell line (intra-species contamination), and can be used to compare to profile databases, which we strongly recommend. This STR report does not rule out cross-contamination with an inter-species cell line (non-human).
- Please see the Pdf file *Additional Information* sent with the results for more information about interpreting your STR profile and links to profile databases.

GRCF DNA Services are for research purposes only. Any data or samples generated by the facility may not be used for human diagnostic or therapeutic use. Results may not be returned to patients, placed into medical records, or be used to make clinical decisions.

Laura Kasch
Johns Hopkins University
2760 Light House Point East, Suite 201
Baltimore, MD 21224
Email: lkasch@jhmi.edu
https://grcf.jhmi.edu/

Principal Investigator: Yulan Cheng
Lab Group: Meltzer
Institution: Johns Hopkins University, GI
Cell Line ID: oe33
Report Date: November 5, 2018

Procedure:

A Promega GenePrint 10 Kit is used to PCR amplify eight short tandem repeat (STR) loci plus a gender determining marker, Amelogenin. The PCR product is electrophoresed on an ABI Prism® 3730xl Genetic Analyzer using an ILS600 internal lane standard (Promega). Data is analyzed using GeneMapper® v 4.0 software (Applied Biosystems). An allelic ladder that contains fragments of the same lengths as many of the known alleles for the loci is used to determine the amplified sample fragments alleles. Appropriate positive and negative controls are used.

Raw Data Table:

Sample Name	Marker	Allele 1	Allele 2	Size 1	Size 2	Height 1	Height 2	Peak Area 1	Peak Area 2
oe33	AMEL	X	X	103.99	103.99	2784	2784	18559	18559
oe33	CSF1PO	10	11	333.51	337.5	2723	797	20953	6329
oe33	D13S317	14	14	199.73	199.73	1170	1170	8029	8029
oe33	D16S539	12	12	290.77	290.77	3660	3660	27055	27055
oe33	D21S11	29	31.2	219.33	229.28	1203	475	7775	2746
oe33	D5S818	11	11	130.44	130.44	2903	2903	20127	20127
oe33	D7S820	9	10	224.11	228.12	1548	4462	10807	32043
oe33	TH01	7	8	165.77	169.73	1702	740	10483	4457
oe33	TPOX	8	11	268.96	280.9	1977	1025	14157	7436
oe33	vWA	14	17	138.35	150.22	626	2861	4585	19209

GRCF DNA Services are for research purposes only. Any data or samples generated by the facility may not be used for human diagnostic or therapeutic use. Results may not be returned to patients, placed into medical records, or be used to make clinical decisions.

grcf at Johns Hopkins
DNA Services/FAF

Laura Kasch
Johns Hopkins University
2760 Light House Point East, Suite 201
Baltimore, MD 21224
Email: lkasch@jhmi.edu
https://grcf.jhmi.edu/

JOHNS HOPKINS MEDICINE

Cell Line Short Tandem Repeat (STR) Profile Report

Principal Investigator: Yulan Cheng
Lab Group: Meltzer
Institution: Johns Hopkins University, GI
Cell Line ID: skgt4
Report Date: November 5, 2018
Submitting Researcher: Yulan Cheng
Sample Type Submitted: cultured cells
GeneSifter Tracking ID: 150777
Profile Kit Requested: GenePrint 10 (Promega)

STR Profile:

Loci	skgt4
AMEL	X
CSF1PO	11,15
D13S317	9,10
D16S539	12,13
D21S11	31.2,31.2
D5S818	12
D7S820	7,11
TH01	6,9.3
TPOX	8,10
vWA	17,19

» Loci with one allele listed are homozygous for that STR marker.

e-Signature Director of Laboratory:

STR Graphic Profile:

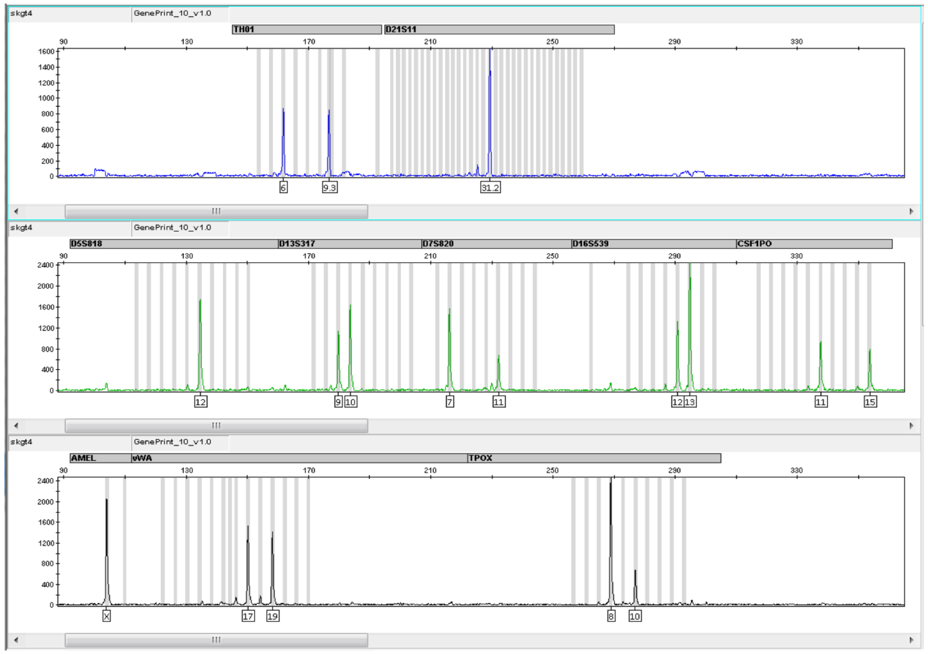

Peaks that appear to be legitimate stutter or are spectral bleed-through artifacts are not labeled.

- This report provides a STR profile for the cell line submitted, it does not automatically authenticate the cell line. Authentication is determined by comparison to the STR profile of the original donor tissue or repository cell stock. This STR profile can be used to verify the cell line is human, evaluate profile consistency between provisionally related cell isolates, detect cross-contamination with another human cell line (intra-species contamination), and can be used to compare to profile databases, which we strongly recommend. This STR report does not rule out cross-contamination with an inter-species cell line (non-human).
- Please see the Pdf file *Additional Information* sent with the results for more information about interpreting your STR profile and links to profile databases.

GRCF DNA Services are for research purposes only. Any data or samples generated by the facility may not be used for human diagnostic or therapeutic use. Results may not be returned to patients, placed into medical records, or be used to make clinical decisions.

grcf at Johns Hopkins DNA Services/FAF
Laura Kasch
Johns Hopkins University
2760 Light House Point East, Suite 201
Baltimore, MD 21224
Email: lkasch@jhmi.edu
https://grcf.jhmi.edu/

JOHNS HOPKINS MEDICINE

Principal Investigator: Yulan Cheng
Lab Group: Meltzer
Institution: Johns Hopkins University, GI
Cell Line ID: skgt4
Report Date: November 5, 2018

Procedure:

A Promega GenePrint 10 Kit is used to PCR amplify eight short tandem repeat (STR) loci plus a gender determining marker, Amelogenin. The PCR product is electrophoresed on an ABI Prism® 3730xl Genetic Analyzer using an ILS600 internal lane standard (Promega). Data is analyzed using GeneMapper® v 4.0 software (Applied Biosystems). An allelic ladder that contains fragments of the same lengths as many of the known alleles for the loci is used to determine the amplified sample fragments alleles. Appropriate positive and negative controls are used.

Raw Data Table:

Sample Name	Marker	Allele 1	Allele 2	Size 1	Size 2	Height 1	Height 2	Peak Area 1	Peak Area 2
skgt4	AMEL	X	X	103.92	103.92	2058	2058	13932	13932
skgt4	CSF1PO	11	15	337.59	353.66	945	796	7918	6361
skgt4	D13S317	9	10	179.82	183.67	1136	1648	8065	11163
skgt4	D16S539	12	13	290.76	294.77	1325	2449	9852	18199
skgt4	D21S11	31.2	31.2	229.29	229.29	1638	1638	9973	9973
skgt4	D5S818	12	12	134.61	134.61	1747	1747	12388	12388
skgt4	D7S820	7	11	216.13	232.24	1572	682	11460	5627
skgt4	TH01	6	9.3	161.83	176.68	871	854	5891	5302
skgt4	TPOX	8	10	268.89	276.84	2478	680	17325	5137
skgt4	vWA	17	19	150.23	158.16	1528	1418	10322	9756

GRCF DNA Services are for research purposes only. Any data or samples generated by the facility may not be used for human diagnostic or therapeutic use. Results may not be returned to patients, placed into medical records, or be used to make clinical decisions.

grcf at Johns Hopkins DNA Services/FAF
Laura Kasch
Johns Hopkins University
2760 Light House Point East, Suite 201
Baltimore, MD 21224
Email: lkasch@jhmi.edu
https://grcf.jhmi.edu/

JOHNS HOPKINS MEDICINE

Cell Line Short Tandem Repeat (STR) Profile Report

Principal Investigator: Yulan Cheng
Lab Group: Meltzer
Institution: Johns Hopkins University
Cell Line ID: JHU-Eso-Ad1
Report Date: January 20, 2020
Submitting Researcher: Yulan Cheng
Sample Type Submitted: Frozen stock
GeneSifter Tracking ID: 163120
Profile Kit Requested: GenePrint 10 (Promega)

STR Profile:

Loci	JHU-Eso-Ad1
AMEL	X
CSF1PO	10
D13S317	11
D16S539	10,12
D21S11	30
D5S818	11
D7S820	10,12
TH01	6,7
TPOX	8,9
vWA	18,19

» Loci with one allele listed are homozygous for that STR marker.

e-Signature Director of Laboratory:

Laura Kasch

Digitally signed by Laura Kasch
DN: cn=Laura Kasch, o=Genetic Resources Core Facility, ou=Johns Hopkins University, email=lkasch@jhmi.edu, c=US
Date: 2020.01.20 09:25:38 -07'00'

STR Graphic Profile:

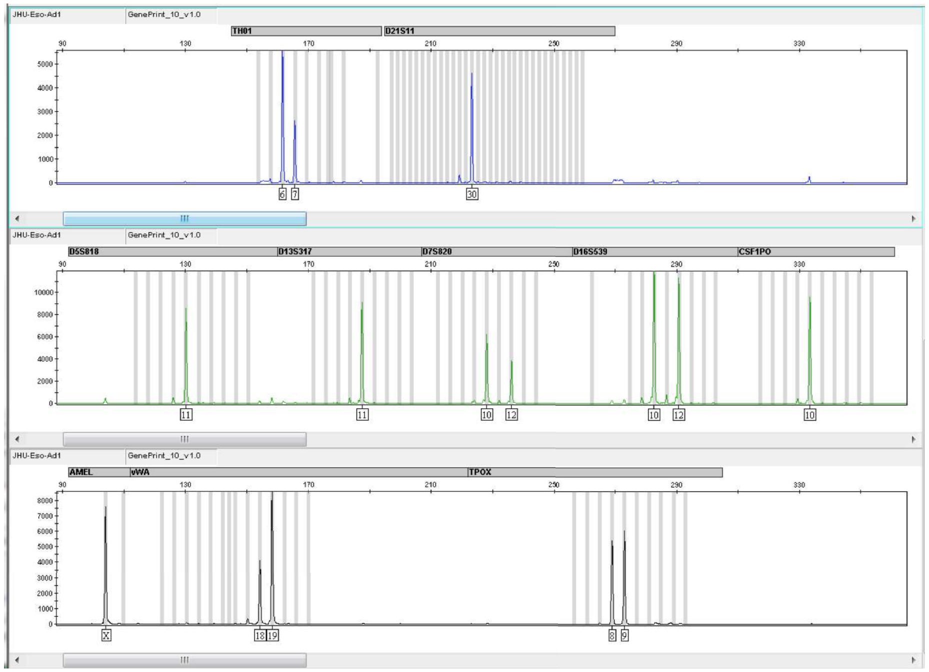

Peaks that appear to be legitimate stutter or are spectral bleed-through artifacts are not labeled.

- This report provides a STR profile for the cell line submitted, it does not automatically authenticate the cell line. Authentication is determined by comparison to the STR profile of the original donor tissue or cell stock. This STR profile can be used to verify the cell line is human, evaluate profile consistency between provisionally related cell isolates, detect cross-contamination with another human cell line (intra-species contamination), and to compare to profile databases, which we strongly recommend. This STR report does not rule out cross-contamination with an inter-species cell line (non-human).
- Please see the Pdf file *Additional Information* sent with the results for more information about interpreting your STR profile and links to profile databases.

GRCF DNA Services are for research purposes only. Any data or samples generated by the facility may not be used for human diagnostic or therapeutic use. Results may not be returned to patients, placed into medical records, or be used to make clinical decisions.

Laura Kasch
Johns Hopkins University
2760 Light House Point East, Suite 201
Baltimore, MD 21224
Email: lkasch@jhmi.edu
https://grcf.jhmi.edu/

Principal Investigator:
Institution:
Cell Line ID:
Report Date:

Procedure:

A Promega GenePrint 10 Kit is used to PCR amplify eight short tandem repeat (STR) loci plus a gender determining marker, Amelogenin. The PCR product is electrophoresed on an ABI Prism® 3730xl Genetic Analyzer using an ILS600 internal lane standard (Promega). Data is analyzed using GeneMapper® v 4.0 software (Applied Biosystems). An allelic ladder that contains fragments of the same lengths as many of the known alleles for the loci is used to determine the amplified sample fragments alleles. Appropriate positive and negative controls are used.

Raw Data Table:

Sample Name	Marker	Allele 1	Allele 2	Size 1	Size 2	Height 1	Height 2	Peak Area 1	Peak Area 2
JHU-Eso-Ad1	AMEL	X	X	103.98	103.98	7566	7566	47267	47267
JHU-Eso-Ad1	CSF1PO	10	10	333.48	333.48	9599	9599	69010	69010
JHU-Eso-Ad1	D13S317	11	11	187.5	187.5	9102	9102	57229	57229
JHU-Eso-Ad1	D16S539	10	12	282.54	290.59	11945	11337	83168	80503
JHU-Eso-Ad1	D21S11	30	30	223.23	223.23	4628	4628	29239	29239
JHU-Eso-Ad1	D5S818	11	11	130.18	130.18	8591	8591	53693	53693
JHU-Eso-Ad1	D7S820	10	12	228.02	236.09	6203	3852	41002	25405
JHU-Eso-Ad1	TH01	6	7	161.61	165.63	5593	2631	34300	16276
JHU-Eso-Ad1	TPOX	8	9	268.95	272.9	5399	6063	36219	41175
JHU-Eso-Ad1	vWA	18	19	154.21	158.19	4104	8578	27072	56406

GRCF DNA Services are for research purposes only. Any data or samples generated by the facility may not be used for human diagnostic or therapeutic use. Results may not be returned to patients, placed into medical records, or be used to make clinical decisions.

grcf at Johns Hopkins DNA Services/FAF

Laura Kasch
Johns Hopkins University
2760 Light House Point East, Suite 201
Baltimore, MD 21224
Email: lkasch@jhmi.edu
https://grcf.jhmi.edu/

JOHNS HOPKINS MEDICINE

Cell Line Short Tandem Repeat (STR) Profile Report

Principal Investigator: Yulan Cheng
Lab Group: Meltzer
Institution: JHU
Cell Line ID: Flo-1
Report Date: October 21, 2019
Submitting Researcher: Yulan Cheng
Sample Type Submitted: Cultured cells, frozen stock
GeneSifter Tracking ID: 160766
Profile Kit Requested: GenePrint 10 (Promega)

STR Profile:

Loci	Flo_1
AMEL	X
CSF1PO	11
D13S317	11
D16S539	12,13
D21S11	30,32.2
D5S818	12,14
D7S820	8
TH01	6
TPOX	9,11
vWA	16

» Loci with one allele listed are homozygous for that STR marker.

e-Signature Director of Laboratory:

Laura Kasch

Digitally signed by Laura Kasch
DN: cn=Laura Kasch, o=JHU GRCF, ou=FAF, email=lkasch@jhmi.edu, c=US
Date: 2019.10.21 13:47:11 -04'00'

STR Graphic Profile:

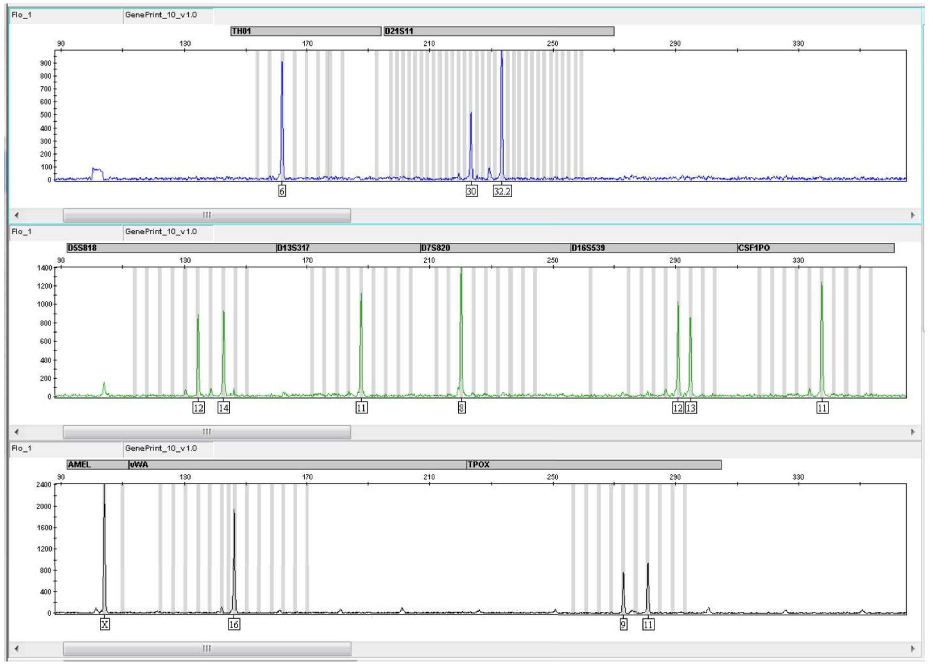

Peaks that appear to be legitimate stutter or are spectral bleed-through artifacts are not labeled.

- This report provides a STR profile for the cell line submitted, it does not automatically authenticate the cell line. Authentication is determined by comparison to the STR profile of the original donor tissue or cell stock. This STR profile can be used to verify the cell line is human, evaluate profile consistency between provisionally related cell isolates, detect cross-contamination with another human cell line (intra-species contamination), and to compare to profile databases, which we strongly recommend. This STR report does not rule out cross-contamination with an inter-species cell line (non-human).
- Please see the Pdf file *Additional Information* sent with the results for more information about interpreting your STR profile and links to profile databases.

GRCF DNA Services are for research purposes only. Any data or samples generated by the facility may not be used for human diagnostic or therapeutic use. Results may not be returned to patients, placed into medical records, or be used to make clinical decisions.

grcf at Johns Hopkins
DNA Services/FAF

Laura Kasch
Johns Hopkins University
2760 Light House Point East, Suite 201
Baltimore, MD 21224
Email: lkasch@jhmi.edu
https://grcf.jhmi.edu/

JOHNS HOPKINS
MEDICINE

Principal Investigator: Yulan Cheng
Lab Group: Meltzer
Institution: JHU
Cell Line ID: Flo-1
Report Date: October 21, 2019

Procedure:

A Promega GenePrint 10 Kit is used to PCR amplify eight short tandem repeat (STR) loci plus a gender determining marker, Amelogenin. The PCR product is electrophoresed on an ABI Prism® 3730xl Genetic Analyzer using an ILS600 internal lane standard (Promega). Data is analyzed using GeneMapper® v 4.0 software (Applied Biosystems). An allelic ladder that contains fragments of the same lengths as many of the known alleles for the loci is used to determine the amplified sample fragments alleles. Appropriate positive and negative controls are used.

Raw Data Table:

Sample Name	Marker	Allele 1	Allele 2	Size 1	Size 2	Height 1	Height 2	Peak Area 1	Peak Area 2
Flo_1	AMEL	X	X	104	104	2421	2421	14324	14324
Flo_1	CSF1PO	11	11	337.55	337.55	1240	1240	9146	9146
Flo_1	D13S317	11	11	187.6	187.6	1122	1122	7228	7228
Flo_1	D16S539	12	13	290.73	294.78	1032	860	7842	6292
Flo_1	D21S11	30	32.2	223.34	233.3	521	997	3404	6374
Flo_1	D5S818	12	14	134.5	142.77	889	932	5388	5935
Flo_1	D7S820	8	8	220.11	220.11	1406	1406	9343	9343
Flo_1	TH01	6	6	161.68	161.68	910	910	5805	5805
Flo_1	TPOX	9	11	272.98	280.98	766	938	5126	6518
Flo_1	vWA	16	16	146.19	146.19	1940	1940	12265	12265

GRCF DNA Services are for research purposes only. Any data or samples generated by the facility may not be used for human diagnostic or therapeutic use. Results may not be returned to patients, placed into medical records, or be used to make clinical decisions.

Laura Kasch
Johns Hopkins University
2760 Light House Point East, Suite 201
Baltimore, MD 21224
Email: lkasch@jhmi.edu
https://grcf.jhmi.edu/

Cell Line Short Tandem Repeat (STR) Profile Report

Principal Investigator: Yulan Cheng
Lab Group: Meltzer
Institution: Johns Hopkins University
Cell Line ID: QHTRT new
Report Date: January 27, 2020
Submitting Researcher: Yulan Cheng
Sample Type Submitted: Cultured cells
GeneSifter Tracking ID: 163364
Profile Kit Requested: GenePrint 10 (Promega)

STR Profile:

Loci	QHTRT new
AMEL	X,Y
CSF1PO	12
D13S317	12,14
D16S539	12,13
D21S11	27,31.2
D5S818	11,12
D7S820	7,8
TH01	7,9.3
TPOX	8,9
vWA	17

» Loci with one allele listed are homozygous for that STR marker.

e-Signature Director of Laboratory:

STR Graphic Profile:

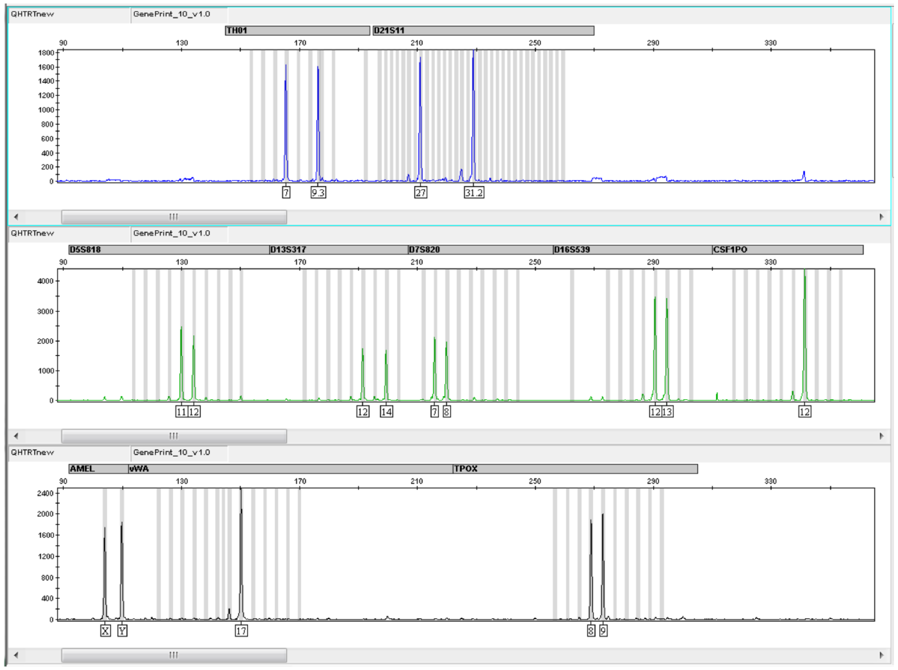

Peaks that appear to be legitimate stutter or are spectral bleed-through artifacts are not labeled.

- This report provides a STR profile for the cell line submitted, it does not automatically authenticate the cell line. Authentication is determined by comparison to the STR profile of the original donor tissue or cell stock. This STR profile can be used to verify the cell line is human, evaluate profile consistency between provisionally related cell isolates, detect cross-contamination with another human cell line (intra-species contamination), and to compare to profile databases, which we strongly recommend. This STR report does not rule out cross-contamination with an inter-species cell line (non-human).
- Please see the Pdf file *Additional Information* sent with the results for more information about interpreting your STR profile and links to profile databases.

GRCF DNA Services are for research purposes only. Any data or samples generated by the facility may not be used for human diagnostic or therapeutic use. Results may not be returned to patients, placed into medical records, or be used to make clinical decisions.

grcf at Johns Hopkins
DNA Services/FAF

Laura Kasch
Johns Hopkins University
2760 Light House Point East, Suite 201
Baltimore, MD 21224
Email: lkasch@jhmi.edu
https://grcf.jhmi.edu/

JOHNS HOPKINS MEDICINE

Principal Investigator: Yulan Cheng
Lab Group: Meltzer
Institution: Johns Hopkins University
Cell Line ID: QHTRT new
Report Date: January 27, 2020

Procedure:

A Promega GenePrint 10 Kit is used to PCR amplify eight short tandem repeat (STR) loci plus a gender determining marker, Amelogenin. The PCR product is electrophoresed on an ABI Prism® 3730xl Genetic Analyzer using an ILS600 internal lane standard (Promega). Data is analyzed using GeneMapper® v 4.0 software (Applied Biosystems). An allelic ladder that contains fragments of the same lengths as many of the known alleles for the loci is used to determine the amplified sample fragments alleles. Appropriate positive and negative controls are used.

Raw Data Table:

Sample Name	Marker	Allele 1	Allele 2	Size 1	Size 2	Height 1	Height 2	Peak Area 1	Peak Area 2
QHTRT new	AMEL	X	Y	104.02	109.79	1742	1845	10995	11403
QHTRT new	CSF1PO	12	12	341.3	341.3	4418	4418	33657	33657
QHTRT new	D13S317	12	14	191.38	199.36	1742	1698	11220	11063
QHTRT new	D16S539	12	13	290.51	294.49	3481	3429	25842	24940
QHTRT new	D21S11	27	31.2	210.89	228.87	1729	1839	11207	12139
QHTRT new	D5S818	11	12	129.91	134.12	2481	2185	15885	13939
QHTRT new	D7S820	7	8	215.83	219.87	2132	1977	14354	13474
QHTRT new	TH01	7	9.3	165.37	176.24	1630	1603	10326	10142
QHTRT new	TPOX	8	9	268.86	272.89	1887	2003	12986	13969
QHTRT new	vWA	17	17	150.16	150.16	2495	2495	16663	16663

GRCF DNA Services are for research purposes only. Any data or samples generated by the facility may not be used for human diagnostic or therapeutic use. Results may not be returned to patients, placed into medical records, or be used to make clinical decisions.

grcf at Johns Hopkins
DNA Services/FAF

Laura Kasch
Johns Hopkins University
2760 Light House Point East, Suite 201
Baltimore, MD 21224
Email: lkasch@jhmi.edu
https://grcf.jhmi.edu/

JOHNS HOPKINS MEDICINE

Cell Line Short Tandem Repeat (STR) Profile Report

Principal Investigator: Yulan Cheng
Lab Group: Meltzer
Institution: Johns Hopkins University
Cell Line ID: GiHTRT new
Report Date: January 27, 2020
Submitting Researcher: Yulan Cheng
Sample Type Submitted: Cultured cells
GeneSifter Tracking ID: 163364
Profile Kit Requested: GenePrint 10 (Promega)

STR Profile:

Loci	GiHTRT new
AMEL	X,Y
CSF1PO	9,13
D13S317	8,10
D16S539	11,12
D21S11	31.2
D5S818	12
D7S820	8,11
TH01	8,9.3
TPOX	8
vWA	17

» Loci with one allele listed are homozygous for that STR marker.

e-Signature Director of Laboratory:

STR Graphic Profile:

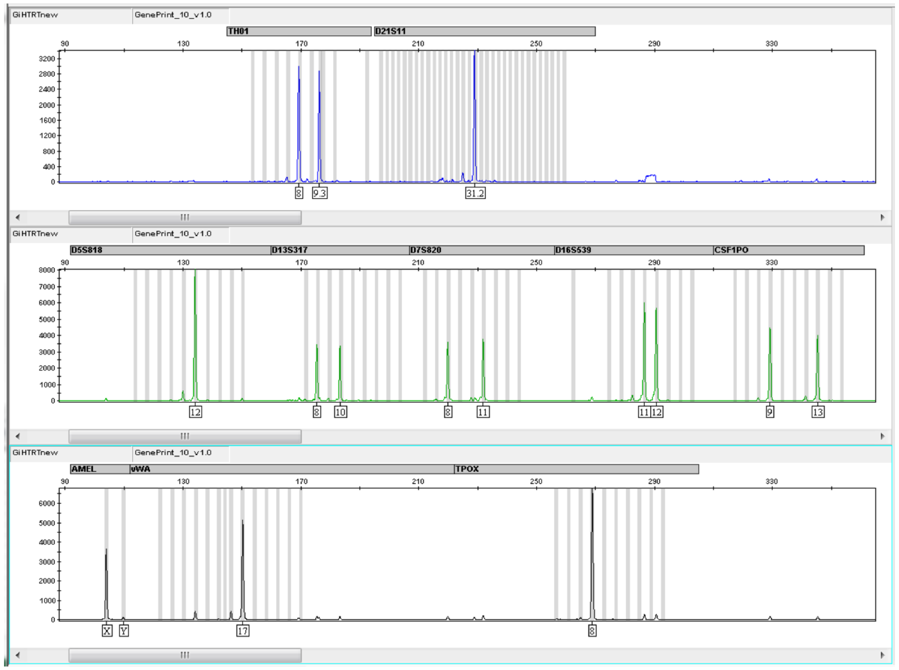

Peaks that appear to be legitimate stutter or are spectral bleed-through artifacts are not labeled.

- This report provides a STR profile for the cell line submitted, it does not automatically authenticate the cell line. Authentication is determined by comparison to the STR profile of the original donor tissue or cell stock. This STR profile can be used to verify the cell line is human, evaluate profile consistency between provisionally related cell isolates, detect cross-contamination with another human cell line (intra-species contamination), and to compare to profile databases, which we strongly recommend. This STR report does not rule out cross-contamination with an inter-species cell line (non-human).
- Please see the Pdf file *Additional Information* sent with the results for more information about interpreting your STR profile and links to profile databases.

GRCF DNA Services are for research purposes only. Any data or samples generated by the facility may not be used for human diagnostic or therapeutic use. Results may not be returned to patients, placed into medical records, or be used to make clinical decisions.

grcf at Johns Hopkins
DNA Services/FAF

Laura Kasch
Johns Hopkins University
2760 Light House Point East, Suite 201
Baltimore, MD 21224
Email: lkasch@jhmi.edu
https://grcf.jhmi.edu/

JOHNS HOPKINS
MEDICINE

Principal Investigator: Yulan Cheng
Lab Group: Meltzer
Institution: Johns Hopkins University
Cell Line ID: GiHTRT new
Report Date: January 27, 2020

Procedure:

A Promega GenePrint 10 Kit is used to PCR amplify eight short tandem repeat (STR) loci plus a gender determining marker, Amelogenin. The PCR product is electrophoresed on an ABI Prism® 3730xl Genetic Analyzer using an ILS600 internal lane standard (Promega). Data is analyzed using GeneMapper® v 4.0 software (Applied Biosystems). An allelic ladder that contains fragments of the same lengths as many of the known alleles for the loci is used to determine the amplified sample fragments alleles. Appropriate positive and negative controls are used.

Raw Data Table:

Sample Name	Marker	Allele 1	Allele 2	Size 1	Size 2	Height 1	Height 2	Peak Area 1	Peak Area 2
GiHTRT new	AMEL	X	Y	103.94	109.79	3636	115	23006	747
GiHTRT new	CSF1PO	9	13	329.12	345.25	4470	4013	32633	30135
GiHTRT new	D13S317	8	10	175.43	183.24	3472	3359	22531	21405
GiHTRT new	D16S539	11	12	286.46	290.54	5988	5669	42966	41229
GiHTRT new	D21S11	31.2	31.2	228.89	228.89	3411	3411	22516	22516
GiHTRT new	D5S818	12	12	134.09	134.09	8049	8049	51597	51597
GiHTRT new	D7S820	8	11	219.85	231.88	3584	3793	24208	25653
GiHTRT new	TH01	8	9.3	169.31	176.23	3005	2880	19799	18071
GiHTRT new	TPOX	8	8	268.89	268.89	6782	6782	46877	46877
GiHTRT new	vWA	17	17	150.27	150.27	5153	5153	34251	34251

GRCF DNA Services are for research purposes only. Any data or samples generated by the facility may not be used for human diagnostic or therapeutic use. Results may not be returned to patients, placed into medical records, or be used to make clinical decisions.

Supplementary Sources

Sequence and characteristics of the SHH promoter region (1kb) and its 5'utr

Cccactcgcctgcccccccccaacaccctgcaatggcctccacacacccagcgcacacactggcccccactctctcccctgtgctggg
gtgcagcccccagacctgctttgcccatccaccctgacgcccgcagttcactctgtaactgcccttgagcacgcctccatcccatttgcagag
ctctacaaaaaaaaaaatgtattaattgaagcacaataaaagagcagtatttttacatttttctaaggcacttaaagcatttcaaataccagtgcttt
aagttatggtcactcataattatcctcggtccacagaggctgagtcttcaggaggggctcagtcctctttcttcaaaatgcagggagagccag
cctgcactataaagtccctggggaagaaaaattaaataacagctgggcagctgtgcccctcaaatgtgtgagcgactctgtgcttgatgactg
aagcttgcgggggtttaaactgtcctttaaaaacttaagttggttttaactgaagcacctaccattccagggaaagtcagggggaaacgcaag
gatgtacacaaagccgggagtaactgctgttaccacaagacatgcgctcctccctcccgcccacctttatcttaggaaagattttttttttttaac
tcgaatccttcaggtgaaaataaagcaacagcagcaacagaaaaaaaaaacgtagtcttcttcagggttaacatcagaagacaagcttgtgc
ggctttggccaatcagatgcgcccctggcagctataatatagtgctaaaggcagcctctctcacaagctctccaggcttgctaccatttaaaat
cagactctttttgtcttttgattgctgtctcgcgacccaactccgatgtgttccgttatcagcggccggcagcctgccattccagcccctgtctgg
gtggggagcggtggagagtcccccgcagccgcggcgggcaaggttatataggaagagaaagaGCGAGGCAGCCAGCG
AGGGAGAGAGCGAGCGGGCGAGCCGGAGCGAGGAAGGGAAAGCGCAAGAGAGAG
CGCACACGCACACACCCGCCGCGCGCACTCGCGCACGGACCCGCACGGGGACAGCT
CGGAAGTCATCAGTTCCATGGGCGAG

SHH Chip F1

OLIGO start len tm gc% any 3' seq

LEFT PRIMER 437 20 60.06 55.00 4.00 2.00 atgtgtgagcgactctgtgc

RIGHT PRIMER 576 20 59.99 50.00 6.00 0.00 ccggctttgtgtacatcctt

SEQUENCE SIZE: 1000

INCLUDED REGION SIZE: 1000

PRODUCT SIZE: 140, PAIR ANY COMPL: 4.00, PAIR 3' COMPL: 0.00

1 LEFT PRIMER 351 20 59.96 50.00 4.00 2.00 cttcaaaatgcagggagagc

RIGHT PRIMER 456 20 60.06 55.00 4.00 2.00 gcacagagtcgctcacacat

PRODUCT SIZE: 106, PAIR ANY COMPL: 4.00, PAIR 3' COMPL: 3.00

OLIGO start len tm gc% any 3' seq

LEFT PRIMER 127 20 60.18 45.00 3.00 0.00 gcaacagcagcaacagaaaa

RIGHT PRIMER 256 20 59.86 55.00 7.00 2.00 gagagaggctgcctttagca

SEQUENCE SIZE: 446

INCLUDED REGION SIZE: 446

PRODUCT SIZE: 130, PAIR ANY COMPL: 5.00, PAIR 3' COMPL: 0.00

Figure S1: *miR205HG* overexpression caused a substantial decrease in EAC invasion in FLO-1 (58% reduction; p-value <0.001) and OE33 (39% reduction; p-value <0.03) cells

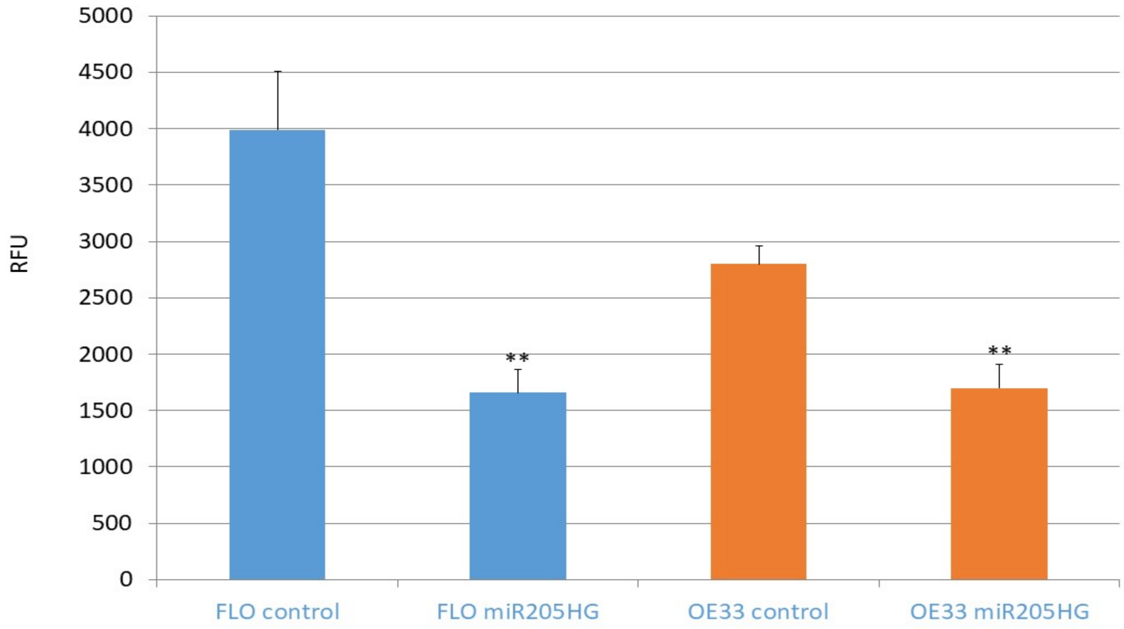

Figure S2. PTCH1 expression in NE-BE tissue pairs. 14 matched NE-BE tissue pairs were assessed for PTCH1 expression by qRT-PCR. PTCH1 was upregulated relative to NE in 13 out of 14 BE tissues studied (average fold-change 23.5, paired t-test p-value <0.0001). (This is the same tissue pairs used in Figure 1E)

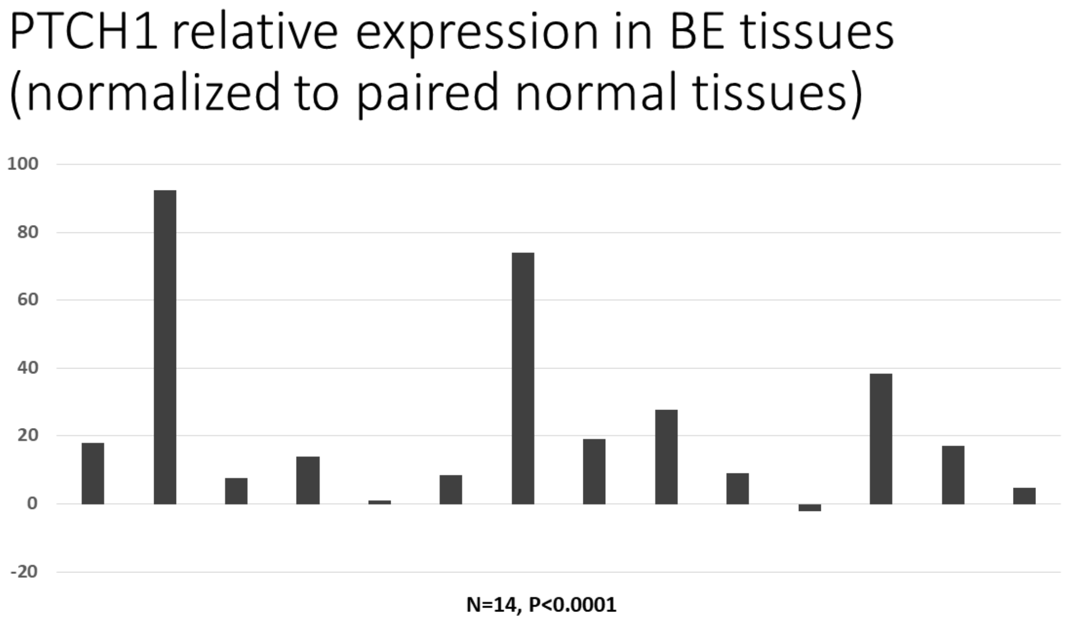

UNCROPPED BLOTS FOR FIGURE 3

200 bp 500bp 1000bp 1500bp 2000bp 3000bp 4000bp

miR205HG

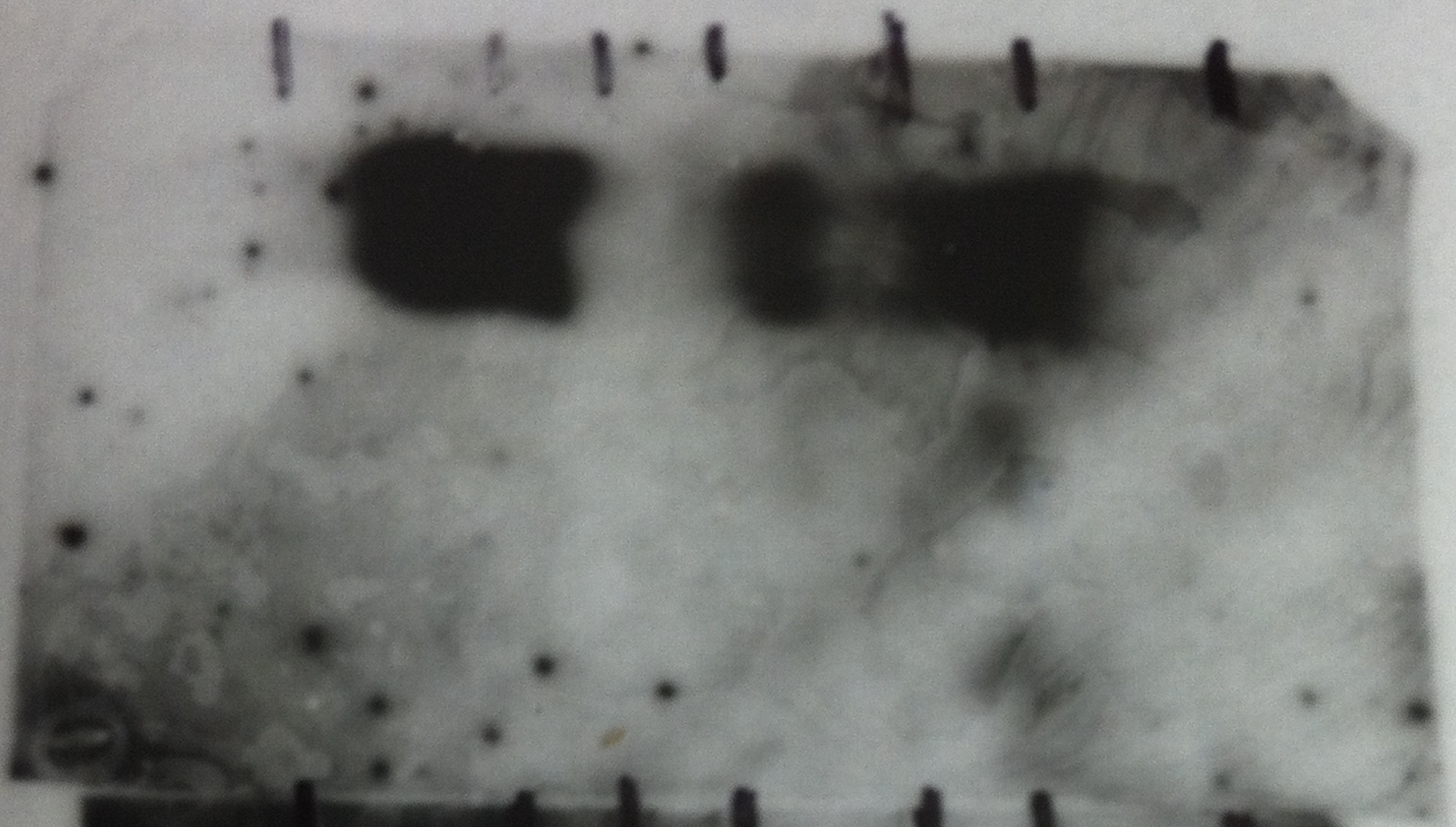

D

Damaged blot -not used

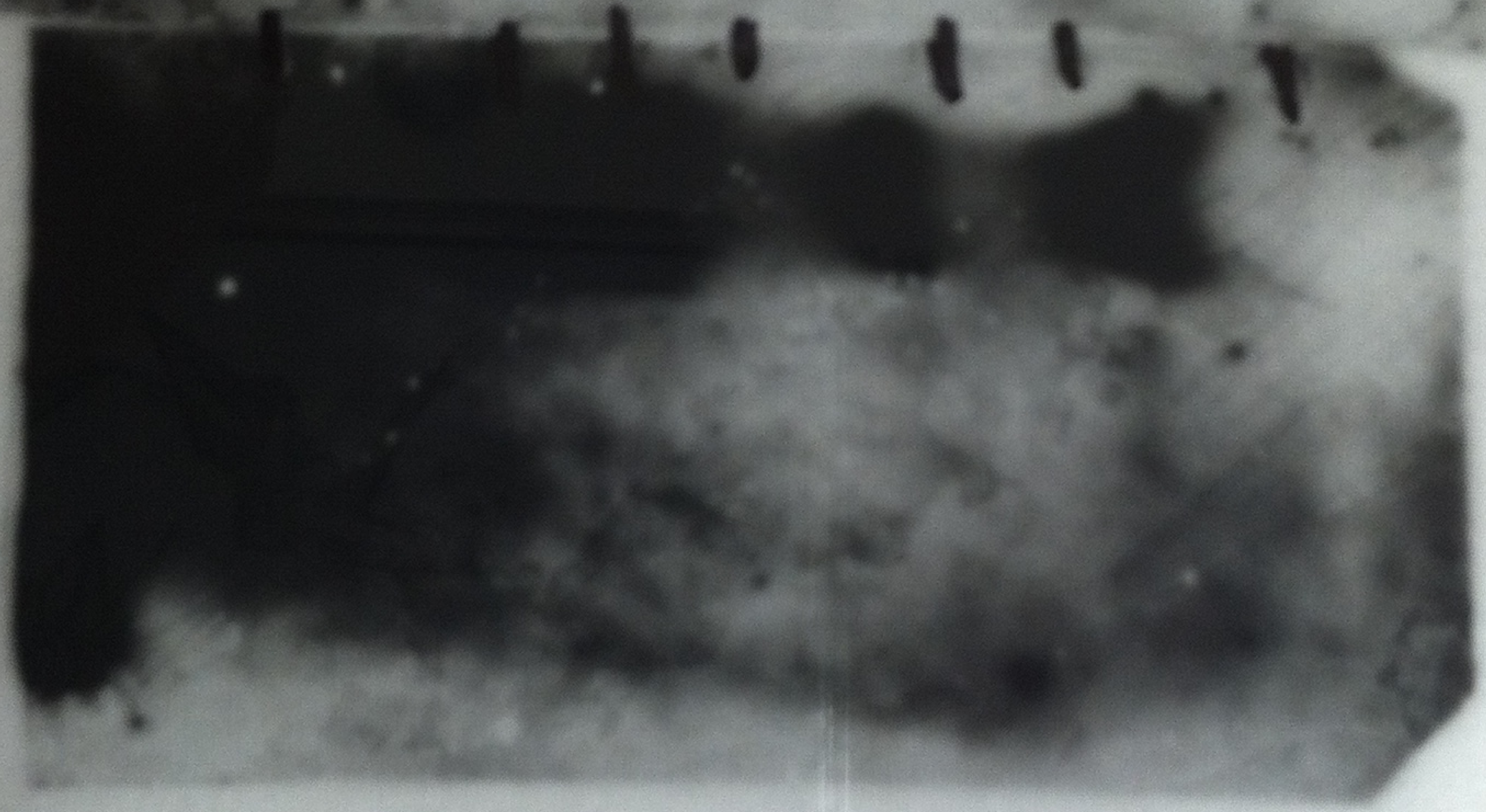

200 bp 500bp 1000bp 1500bp 2000bp 3000bp 4000bp

GAPDH

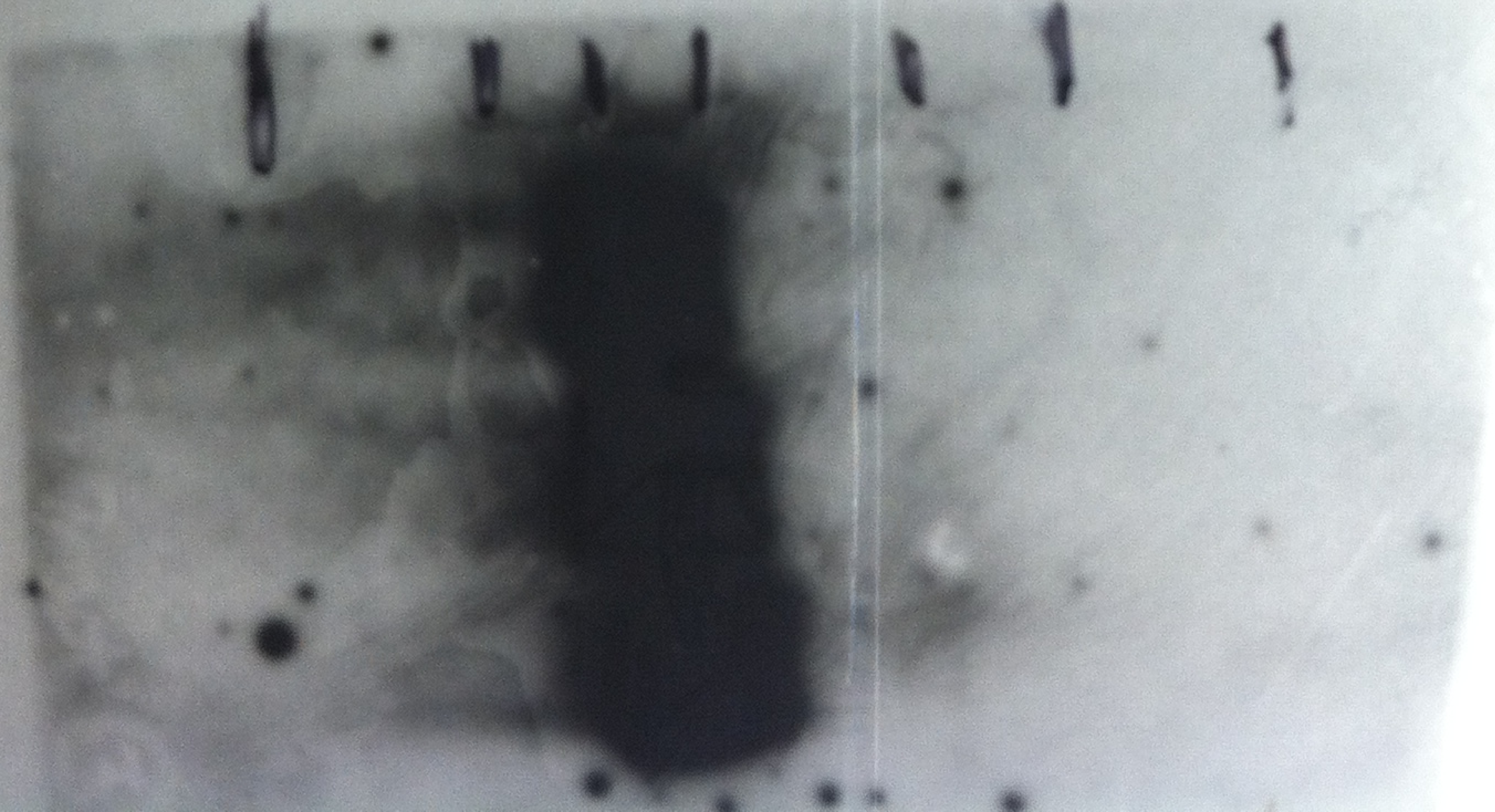

ORIGINAL BLOT FIGURE 18

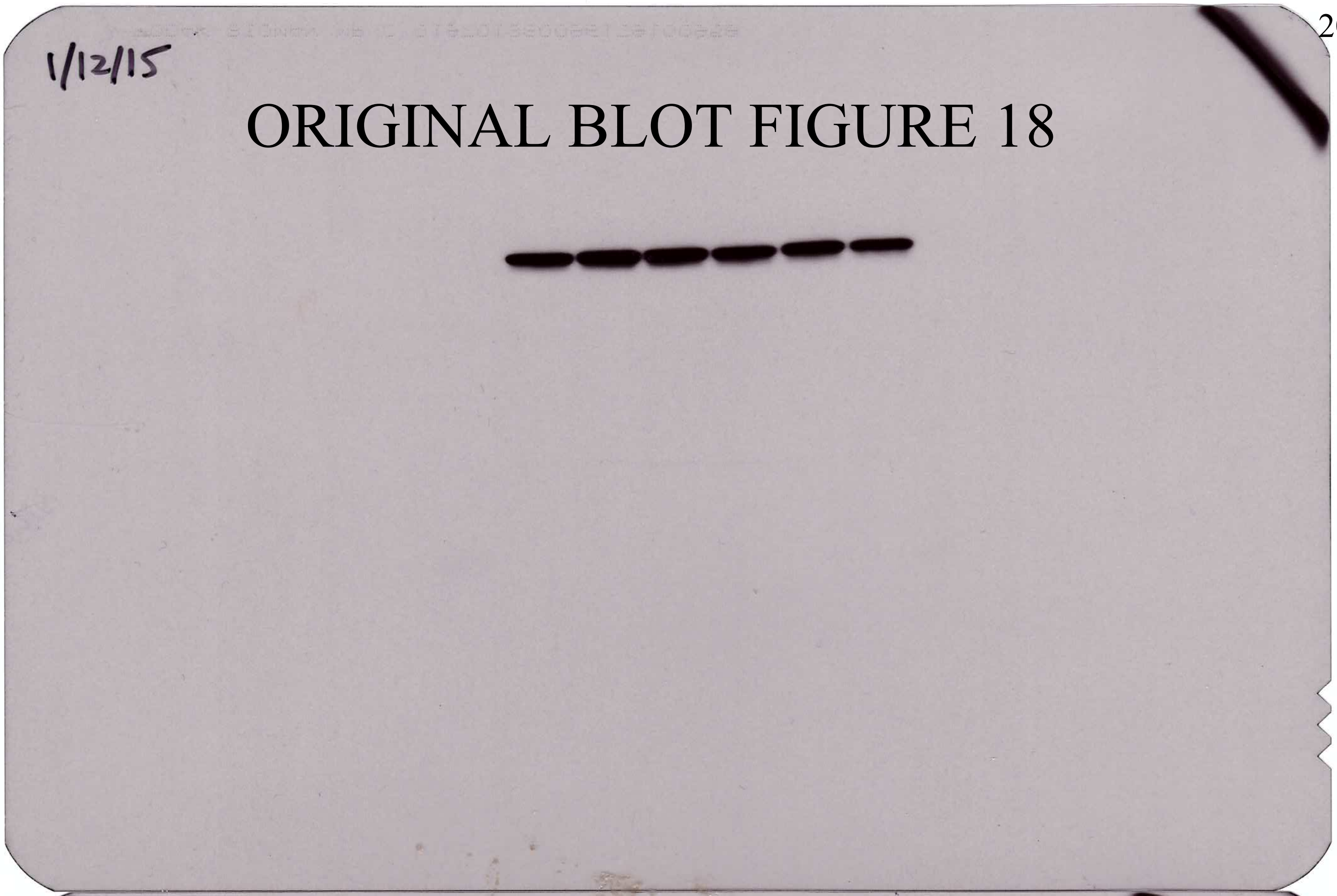

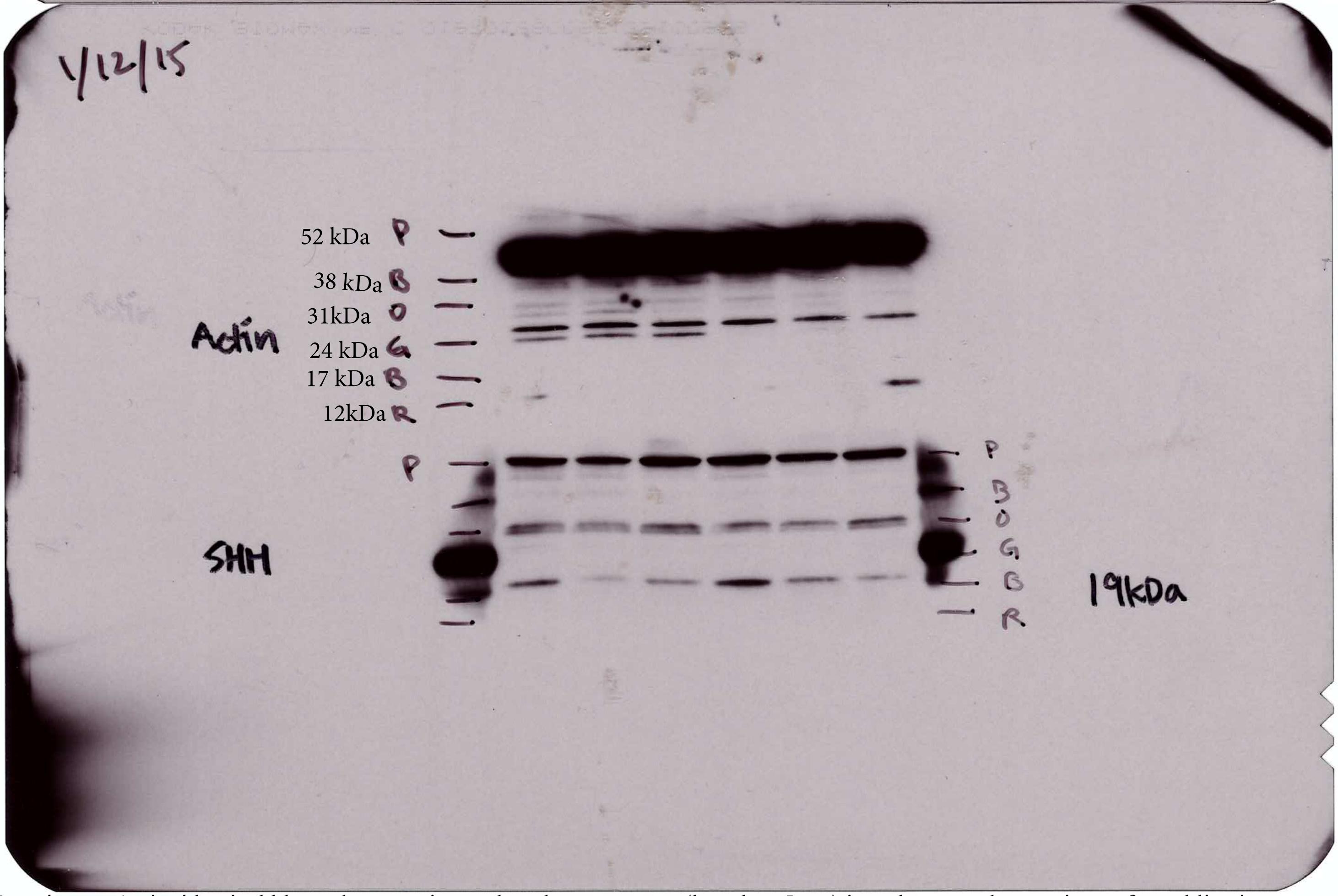

Top picture: Actin, identical blot as bottom picture, but short exposure (less than 5 sec) in order to get better picture for publication.

Bottom picture: Actin and SHH as labeled. We used Rainbow marker. From the bottom Red (12 kDa), Blue 17, green 24, Orange 31, Blue 38, Purple 52.
